# Spontaneous coronary artery dissection in a patient with autosomal dominant polycystic kidney disease: a case report

**DOI:** 10.1186/s13256-016-0832-8

**Published:** 2016-03-10

**Authors:** Peeyush Grover, Timothy P. Fitzgibbons

**Affiliations:** 1 Department of Medicine, Cardiovascular Division, University of Massachusetts Medical School, Worcester, MA 01605 USA; 2 Cardiovascular Medicine, Umass Memorial Medical Center, Worcester, MA 01605 USA

**Keywords:** Polycystic kidney disease, Right coronary artery, Spontaneous coronary artery dissection

## Abstract

**Background:**

Spontaneous coronary artery dissection is an uncommon syndrome. Its prevalence among patients with polycystic kidney disease is very rare, with no previously reported involvement of the right posterior descending coronary artery.

**Case presentation:**

We describe the case of a middle-aged Caucasian woman with polycystic kidney disease who presented with a non-ST elevation myocardial infarction. Cardiac catheterization revealed a dissection of her right posterior descending coronary artery. She was treated with dual antiplatelet therapy and had a favorable outcome.

**Conclusion:**

We report a rare and interesting case of spontaneous coronary artery dissection of the right posterior descending coronary artery in a patient with polycystic kidney disease. It is important to consider spontaneous coronary artery dissection in the differential diagnosis of patients with polycystic kidney disease who present with an acute coronary syndrome.

## Background

Spontaneous coronary artery dissection (SCAD) is a known cause of acute coronary syndrome [1–4]. It has been reported to present in peripartum women, in patients with a history of fibromuscular dysplasia or inherited collagen vascular disorders, and with the use of hormonal therapy [5]. The association of SCAD with autosomal dominant polycystic kidney disease (PKD) is less well recognized and limited to only a handful of cases [6–11]. Our reported case is of a woman with PKD who presented with SCAD of her right posterior descending coronary artery (RPDA). Our patient did not need any acute interventions and had spontaneous resolution of symptoms with no recurrent episodes reported over a 1-year follow-up. This is a rare and interesting presentation of an uncommon disorder.

## Case presentation

A 52-year-old Caucasian woman with a known history of autosomal dominant PKD presented with acute onset chest pain lasting over 1 day. She had no prior history of coronary disease or diabetes, nor a personal or family history of connective tissue disorder or vasculitis, and did not use oral contraceptives. The pain was sub-sternal in location with radiation to her left arm and was reported as pressure-like in quality. It started while she was sitting and improved with the use of sublingual nitroglycerine on initial presentation to our emergency department. She also reported transient left-sided facial droop at the start of her symptoms. Her symptoms lasted only for a few minutes and spontaneously resolved.

**Fig. 1:**
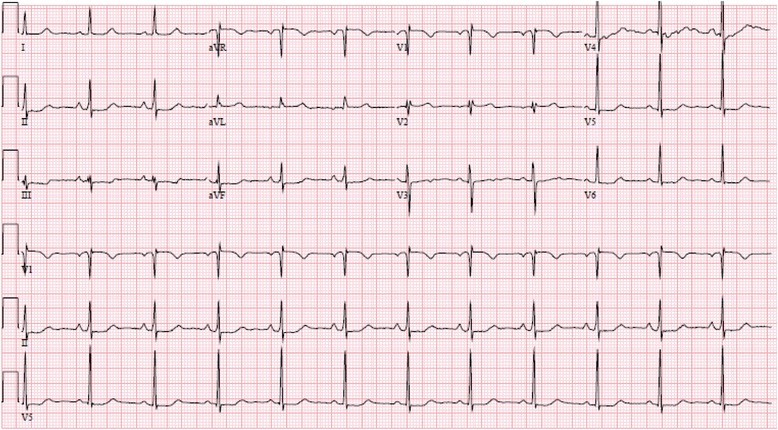
Electrocardiography at the time of presentation to the emergency department demonstrating normal sinus rhythm with non-specific ST and T wave changes in the inferior (II, III, and aVF) and lateral leads (V 4-6)

**Fig. 2:**
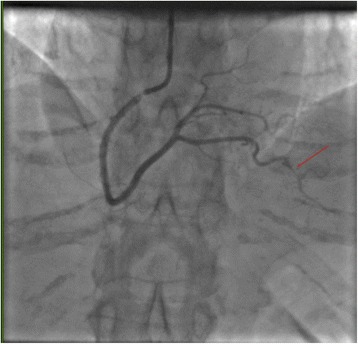
Right anterior oblique view of the right coronary artery demonstrating the spontaneous coronary artery dissection at the distal end (the culprit for our patient’s presentation) of the right posterior descending artery

An initial electrocardiogram demonstrated normal sinus rhythm with nonspecific ST–T wave changes in the inferior leads (Fig. 1). Cardiac enzymes (troponin I) demonstrated a rising trend (peak 3.14 U/ml) during the day of admission. A non-contrast computed tomography (CT) scan of her brain demonstrated no acute bleed. No atherosclerotic disease was identified via CT angiography of her head and neck vessels. Magnetic resonance imaging (MRI) of her brain with contrast was also performed. This showed no dissection of her intracranial vessels, no cerebral aneurysms, and no evidence of stroke. No vertebral or carotid artery dissection was seen on these studies. An echocardiogram demonstrated hypokinesis of the mid- to apical inferior wall and mid-inferolateral wall. She subsequently underwent cardiac catheterization, which demonstrated spontaneous coronary artery dissection of her distal RPDA (Fig. 2). No interventions were performed and our patient was started on dual anti-platelet therapy (DAPT). She was followed up over one year with no reported recurrent symptoms.

## Discussion

We describe a case of spontaneous RPDA dissection in the setting of autosomal dominant PKD. Although previously described in the literature, spontaneous coronary artery dissection continues to be an infrequently reported syndrome. To the best of our knowledge, only six such cases have previously been published [6–11]. None of these reported cases had isolated involvement of the RPDA. Although it is more common in women, SCAD should be considered in the differential diagnosis of all patients with acute coronary syndromes. It may occur in the presence or absence of coronary atherosclerosis [12]. In patients without atherosclerosis, SCAD more commonly involves the left anterior descending artery (67 %), whereas involvement of the right coronary artery is less common (15–30 %). Only one of the patients in these reported cases is mentioned to have a history of PKD [5, 12]. It is unclear from the series whether the reported patient had RPDA involvement or not. Interestingly, SCAD in patients with co-existing coronary atherosclerosis more commonly affects the right coronary artery (50 %) than the left anterior descending artery (33 %) [12]. Although our patient had no angiographic evidence of coronary disease, the sensitivity of angiography alone is limited; therefore, we cannot totally exclude this possibility. More sensitive methods for the detection of coronary disease, such as intra-vascular ultrasound, optical coherence tomography, or CT angiography may have detected non-occlusive plaque.

Some have proposed classification of the risk factors for SCAD into three broad categories: atherosclerosis, pregnancy-associated, and idiopathic [13]. Connective tissue disorders, such as Type IV Ehlers–Danlos syndrome, or in this case, PKD, are included in the idiopathic category. PKD is considered to manifest if there is reduced expression of polycystins (polycystin 1 and 2 due to mutation in genes *PKD1* and *PKD2*, respectively). These protein products are thought to play a crucial role in the coupling between various layers of the tunica media. Thus their normal expression is believed to be crucial for maintaining the integrity of the myoelastic structure of the arterial wall [14]. Owing to its rarity, the prevalence of SCAD in patients with PKD is currently unclear. This case highlights the fact that patients with PKD should be considered at high risk for SCAD even if they have no coronary artery disease risk factors.

The causes of pregnancy-associated SCAD are not entirely understood [15]. The dramatic endocrine and hemodynamic changes that occur in pregnancy are likely to play a role. The precise adverse effects of estrogen and progesterone on the arterial wall are not known, but a pathologic mechanism is supported by multiple lines of evidence: (1) a greater incidence of SCAD in women, (2) a greater incidence in the peripartum period, (3) reports of SCAD during the menstrual cycle, and (4) the known association with oral contraceptive use. Among other possible effects, progesterone or estrogen may stimulate degradation of collagen within the arterial media [15].

Our patient had SCAD in the distal portion of her RPDA. The lesion was not amenable to coronary intervention. She was started on DAPT and did not have any recurrent symptoms. The use of conservative medical therapy to treat patients with SCAD is well reported [1, 5, 12]. When SCAD occurs in a distal vessel not amenable to coronary intervention, and patients are asymptomatic and hemodynamically stable, treatment with DAPT is often sufficient, and allows for spontaneous healing of the vessel wall over time [1, 12].

It is unclear to us why our patient had initial symptoms of a neurological deficit. These symptoms could have been caused by a spontaneous carotid or vertebral vessel dissection that was beyond the sensitivity of the brain MRI scan and CT angiogram. Similar cases have previously been described in the literature [8]. These patients usually have a good prognosis with rarely reported recurrent symptoms [6–11].

## Conclusion

It is important to include SCAD in the differential diagnosis of patients with PKD who present with an acute coronary syndrome.

## Consent

Written informed consent was obtained from the patient for publication of this case report and accompanying images. A copy of the written consent is available for review by the Editor-in-Chief of this journal.
